# Different Profiles of Cytokines, Chemokines and Coagulation Mediators Associated with Severity in Brazilian Patients Infected with Dengue Virus

**DOI:** 10.3390/v13091789

**Published:** 2021-09-08

**Authors:** Victor Edgar Fiestas Solórzano, Nieli Rodrigues da Costa Faria, Caroline Fernandes dos Santos, Gladys Corrêa, Márcio da Costa Cipitelli, Marcos Dornelas Ribeiro, Luiz José de Souza, Paulo Vieira Damasco, Rivaldo Venâncio da Cunha, Flavia Barreto dos Santos, Luzia Maria de Oliveira Pinto, Elzinandes Leal de Azeredo

**Affiliations:** 1 Viral Immunology Laboratory, Instituto Oswaldo Cruz—Fiocruz, Rio de Janeiro 21040-360, Brazil; vicfiso@gmail.com (V.E.F.S.); nielircf@gmail.com (N.R.d.C.F.); caroline.santos@ioc.fiocruz.br (C.F.d.S.); gladys.correa@gmail.com (G.C.); flaviab@ioc.fiocruz.br (F.B.d.S.); lpinto@ioc.fiocruz.br (L.M.d.O.P.); 2 Molecular Biology Laboratory, Instituto de Biologia do Exército, Rio de Janeiro 20911-270, Brazil; marcio_cipitelli@msn.com (M.d.C.C.); dornelas-ribeiro@hotmail.com (M.D.R.); 3 Centro de Referência de Doenças Imuno-Infecciosas, Campos dos Goytacazes 28025-496, Brazil; luizjosedes@gmail.com; 4 Departamento de Clínica Médica, Faculdade de Medicina de Campos, Campos dos Goytacazes 28035-581, Brazil; 5 Rede Casa Hospital Rio Laranjeiras, Rio de Janeiro 22240-000, Brazil; paulovieiradamasco@gmail.com; 6 Faculdade de Ciências Médicas, Universidade do Estado do Rio de Janeiro, Rio de Janeiro 20550-170, Brazil; 7 Escola de Medicina e Cirurgia, Universidade Federal do Estado do Rio de Janeiro, Rio de Janeiro 20270-330, Brazil; 8 Faculdade de Medicina, Universidade Federal de Mato Grosso do Sul, Campo Grande 79070-900, Brazil; rivaldo.cunha@fiocruz.br

**Keywords:** dengue, principal component analysis, biomarkers, Brazil

## Abstract

The incidence of dengue in Latin America has increased dramatically during the last decade. Understanding the pathogenic mechanisms in dengue is crucial for the identification of biomarkers for the triage of patients. We aimed to characterize the profile of cytokines (IFN-γ, TNF-α, IL-1β, IL-6, IL-18 and IL-10), chemokines (CXCL8/IL-8, CCL2/MCP-1 and CXCL10/IP-10) and coagulation mediators (Fibrinogen, D-dimer, Tissue factor-TF, Tissue factor pathway inhibitor-TFPI and Thrombomodulin) during the dengue-4 epidemic in Brazil. Laboratory-confirmed dengue cases had higher levels of TNF-α (*p* < 0.001), IL-6 (*p* = 0.005), IL-10 (*p* < 0.001), IL-18 (*p* = 0.001), CXCL8/IL-8 (*p* < 0.001), CCL2/MCP-1 (*p* < 0.001), CXCL10/IP-10 (*p* = 0.001), fibrinogen (*p* = 0.037), D-dimer (*p* = 0.01) and TFPI (*p* = 0.042) and lower levels of TF (*p* = 0.042) compared to healthy controls. A principal component analysis (PCA) distinguished between two profiles of mediators of inflammation and coagulation: protective (TNF-α, IL-1β and CXCL8/IL-8) and pathological (IL-6, TF and TFPI). Lastly, multivariate logistic regression analysis identified high aspartate aminotransferase-to-platelet ratio index (APRI) as independent risk factors associated with severity (adjusted OR: 1.33; 95% CI 1.03–1.71; *p* = 0.027), the area under the receiver operating characteristics curve (AUC) was 0.775 (95% CI 0.681–0.869) and an optimal cutoff value was 1.4 (sensitivity: 76%; specificity: 79%), so it could be a useful marker for the triage of patients attending primary care centers.

## 1. Introduction

Dengue is a mosquito-borne viral disease caused by the dengue virus (DENV), which belongs to the family *Flaviviridae*, genus *Flavivirus*. There are four different but genetically related serotypes (DENV-1, DENV-2, DENV-3 and DENV-4). Dengue is endemic in more than 100 countries, and it has been estimated that 96 million symptomatic infections occur per year with different degrees of severity [1]. The incidence of dengue in Latin America has increased dramatically during the last decade [2] due to several factors such as population growth, rapid and unplanned urbanization, migration, geographical conditions and barriers to preventive care, with a considerable economic impact on these countries [3].

In the current context of the COVID-19 pandemic, the Americas region in 2020 had a lower cumulative incidence rate of dengue than in the previous year, but this continued to be higher than the incidence rate reported for the 2016–2018 period [4]. Thus, given the evidence of higher-than-expected persistence of dengue cases in endemic areas, the PAHO/WHO has recommended strengthening the triage of suspected dengue cases [4].

Dengue is a dynamic disease. While most patients recover after a self-limited clinical course, some progress to severe illness and death [5]. The clinical classification proposed by the World Health Organization (WHO) in 2009 [6] provides a set of clinical warning signs to help predict severe disease and thus provide early and appropriate treatment for patients. However, not all warning signs appear at the beginning of the disease, so the identification and validation of markers for triage and treatment is still a challenge [7]. Furthermore, there is no specific treatment, and the vaccines still have inconsistent effectiveness against all four dengue serotypes [8].

After the reintroduction of DENV in Brazil in 1982, its spread throughout the country produced recurrent epidemics that were generally associated with the introduction of new serotypes [9]. In 2013, the DENV-4 genotype II was the most prevalent serotype [10,11] in an explosive epidemic that caused a high number of cases and deaths, mainly in the states of the southeastern and central-western regions [12,13]. Although the majority of DENV-4 infections were generally characterized by mild clinical pictures compared to other serotypes [14], severe cases and deaths due to DENV-4 were reported [15]. Thus, the impact that the re-emergence of DENV-4 had in this endemic country is still not well understood, and studies that include the evaluation of the inflammatory response are necessary.

The pathogenic mechanisms through which DENV causes severe disease are not yet fully understood, although it has been established that factors such virus strain and the host’s age and immune and genetic status are fundamental to the response to DENV infection [16]. Secondary infection by a heterologous DENV serotype have been proposed as risk factor for the progression of severe cases, a phenomenon of antibody-dependent enhancement [17]. Extensive evidence suggests that excessive production of inflammatory mediators from immune cells is related to dengue severity [18]. In fact, the activation of innate and adaptive immune responses causes increased released of cytokines, a phenomenon described as a cytokine storm. This phenomenon increases the risk of vascular permeability and multi-organ failure and has been proposed to play an important role in the pathogenesis of severe dengue [5].

Previous studies have shown that levels of cytokines such as interferon gamma (IFN-γ), tumor necrosis factor α (TNF-α) and interleukin-1β (IL-1β), IL-6, IL-18, IL-10 and chemokines such as the C-X-C motif chemokine ligand 8/interleukin-8 (CXCL8/IL-8),the C-C motif chemokine ligand 2/monocyte chemoattractant protein-1 (CCL2/MCP-1) and the C-X-C motif chemokine ligand 10/interferon protein-10 (CXCL10/IP-10) are elevated in DENV infection [19,20,21,22,23,24]. In addition, coagulation mediators are being studied since a better understanding of the crosstalk between activated coagulation and inflammatory response in the pathogenesis of DENV infection is still needed [25,26,27].

The aim of this study was to characterize the profile of cytokines/chemokines and coagulation mediators in order to better understand the events of the inflammatory response that have been correlated with severity in dengue, which could contribute to identification of biomarkers for the adequate triage of patients.

## 2. Materials and Methods

### 2.1. Ethics Statement

This study was conducted in accordance with the principles of the Declaration of Helsinki and was approved by the Research Ethics Committee of the Oswaldo Cruz Institute, Oswaldo Cruz Foundation, Ministry of Health, Brazil (CEP 274/05, CAAE: 13318113.7.0000.5248, 16 December 2013).

### 2.2. Study Population

In the period from January–March 2013, adults who met criteria for a suspected dengue case according to the guidelines of the Brazilian Ministry of Health [28] were enrolled at healthcare centers in the states of Rio de Janeiro (RJ) and Mato Grosso do Sul (MS): Rio Laranjeiras Hospital, RJ; Plantadores de Cana Hospital, RJ; Reference Center for Infectious and Parasitic Diseases (CEDIP), MS; and Guanandy Health Center, MS.

### 2.3. Sample Collection

After the participants gave their informed consent, approximately 20 mL of peripheral blood was obtained by means of venipuncture using BD Vacutainer™ tubes containing acid-citrate-dextrose (catalogue # BD 364606), and the plasma was centrifuged, aliquoted and stored at −70 °C until analysis. In addition, 15 adults who had not had febrile episode in the last three months or any history of other diseases were included as healthy controls for analysis of cytokines, chemokines and coagulation factors.

### 2.4. Dengue Diagnosis

All the dengue patients were positive by molecular and/or serological assays.

#### 2.4.1. Serological Assays

Detection of nonstructural protein 1 (NS1) antigen was performed using the Platelia^TM^ Dengue NS1 Ag ELISA kit (Bio-Rad Laboratories, Marnes La Coquette, France), in accordance with the manufacturer’s instructions. Quantification of circulating DENV NS1 antigen was also performed, as described previously [29]. Detection of immunoglobulin M (IgM) anti-dengue was done using the Panbio^®^ Dengue IgM capture ELISA kit (Panbio Inc., Queensland, Australia), in accordance with the manufacturer’s instructions.

IgG anti-dengue detection by means of the Dengue Virus IgG DxSelect^TM^ kit (Focus Diagnostics, Cypress, CA, USA) was used to differentiate between primary and secondary infection. The infection was established as primary when the IgM/IgG optical density ratio was ≥1.2, as previously described [30].

#### 2.4.2. Molecular Assays

For molecular tests, viral RNA was extracted using the QIAamp Viral RNA Mini kit (Qiagen, Hilden, Germany), in accordance with the manufacturer’s instructions. DENV detection and typing were performed as described previously [31] and by using the Simplexa^TM^ Dengue real-time RT-PCR assay (Focus Diagnostics, Cypress, CA, USA), in accordance with the manufacturer’s instructions.

### 2.5. Classification of Cases

The participants were classified as presenting dengue without warning signs (DwoWS), dengue with warning signs (DwWS) or severe dengue (SD), in accordance with the WHO’s 2009 guidelines [6]. Febrile patients with negative tests for dengue were classified as presenting acute undifferentiated febrile disease (AUFI).

### 2.6. Hematological and Biochemical Parameters

The results from laboratory tests such as complete blood count, aspartate aminotransferase (AST), alanine aminotransferase (ALT), fibrinogen, prothrombin time (PT) and activated partial thromboplastin time (aPTT) performed on admission were collected for analysis in this study. Furthermore, the aspartate aminotransferase-to-platelet ratio index (APRI) was calculated using the following formula: AST (IU/L)/upper limit normal/platelet count (×10^9^/L) × 100. The neutrophil-to-lymphocyte ratio (NLR), monocyte-to-lymphocyte ratio (MLR) and platelet-to-lymphocyte ratio (PLR) were also calculated.

### 2.7. Quantification of Cytokines and Chemokines

Plasma levels of cytokines and chemokines were measured using Luminex^®^ technology and ELISA kits, in accordance with the manufacturer’s instructions. Plasma levels of TNF-α (range: 0.82–3350 pg/mL), IL-1β (range: 0.37–1500 pg/mL), IL-10 (range: 0.78–3200 pg/mL) and CXCL8/IL-8 (range: 0.78–3200 pg/mL) were quantified using a multiplex immunoassay, in accordance with the manufacturer’s instructions (IL-1β cat. LHSC201; CXCL8/IL-8 cat. LHSC208; TNF-α cat. LHSC210; and IL-10 cat. LHSC217, R&D Systems, USA). Plasma levels of IFN-γ (range: 46.8–3000 pg/mL), IL-6 (range: 9.3–600 pg/mL), CCL2/MCP-1 (range: 3.9–250 pg/mL), CXCL10/IP-10 (range: 1.9–125 pg/mL) and IL-18 (range: 3.9–250 pg/mL) were measured using ELISA kits, in accordance with the manufacturer’s instructions: IFN-γ (cat. 900-K27, Peprotech); IL-6 (cat. DY206, R&D Systems); CCL2/ MCP-1 (cat. 900-K31, Peprotech); CXCL10/IP-10 (cat. 900-K39, Peprotech); and IL-18 (cat. RAB0543, Sigma). Standard curves of known concentrations of recombinant human cytokines or chemokines were used to convert optical density (OD) into concentration units (pg/mL). The plates were read in a SpectraMax Paradigm^®^ machine (Molecular Devices, San Jose, CA, USA).

### 2.8. Quantification of Tissue Factor (TF), Tissue Factor Pathway Inhibitor (TFPI), Thrombomodulin (TM) and D-Dimer

Plasma levels of TF (range: 7.8–500 pg/mL), TFPI (range 31.3–2000 pg/mL) and TM (range 62.5–4000 pg/mL) were measured using ELISA kits, in accordance with the manufacturer’s instructions: TF (cat. DCF300, R&D Systems); TFPI (cat. DTFP10, R&D Systems) and TM (cat. DTHBD0, R&D Systems). Standard curves of known concentrations of recombinant human proteins (TF, TFPI and TM) were used to convert optical density (OD) into concentration units (pg/mL). The plates were read in a SpectraMax Paradigm^®^ machine (Molecular Devices, California, USA). Plasma levels of D-dimer were measured using HemosIL D-Dimer HS 500 kit (cat. 0020500100) on an automated coagulation analyzer ACL TOP 300 CTS (Instrumentation Laboratory, Bedford, MA, USA).

### 2.9. Statistical Analysis

The Shapiro–Wilk test was used to assess the normality of the measured data. Continuous variables were presented as means with standard deviations (SDs) or as medians with interquartile ranges (IQRs), as appropriate. The Kruskal–Wallis test (followed by Dunn’s multiple comparisons test), one-way ANOVA, chi-square test and Fisher’s exact test were used to compare differences between groups, as appropriate. Spearman’s rank correlation between the variables studied was represented in a correlogram.

Principal component analysis (PCA) was used to reduce dimensionality and identify cytokines/chemokines and coagulation mediator patterns associated with disease severity. The Kaiser–Meyer–Olkin (KMO) test and Bartlett’s sphericity test were used to assess whether the data were adequate for this analysis. PCA with subsequent varimax rotation determined the number of principal components and the cumulative percentage of variance. According to the Kaiser criterion, we use only principal components with associated eigenvalues greater than one. The individual scores for each component were transformed to a scale from 0 to 1, and the difference between the groups of patients was evaluated.

Bivariate analyses and stepwise multivariate logistic regression with backward elimination (*p* < 0.05) were used to identify laboratory markers associated with severity. The interactions of independent variables were assessed through a multicollinearity test using the variance inflation factor (VIF). The area under the receiver operating characteristic (ROC) curve (AUC) was calculated and the optimal cutoff point was determined using the Youden index.

All statistical analyses were conducted in the R Statistical Language version R-4.04 for Windows [R Core Team (2021). R: A language and environment for statistical computing and R Foundation for Statistical Computing, Vienna, Austria. URL: https://www.r-project.org/ (accessed on 20 April 2021)]; *p* values < 0.05 were considered statistically significant.

## 3. Results

### 3.1. Baseline Clinical Characteristics of the Confirmed Dengue Cases

During the study period, 316 patients were recruited. The diagnosis of dengue was confirmed in 225 patients (71.2%), and 91 patients (28.8%) with negative serological and molecular tests were classified as AUFI. The median age of the dengue patients was 36 years (IQR: 26–50 years), 58% were women (male-to-female ratio = 0.7) and the most frequent comorbidity was hypertension (19%).

Based on the 2009 WHO dengue classification [5], the dengue patients were classified as DwoWS (162/225; 72%) or DwWS/SD (63/225; 28%). There were no statistically significant differences between the dengue patient groups and the AUFI patients in relation to age, gender or comorbidity. DwWS/SD patients had a median number of days with illness (5 days) that was greater than that of the DwoWS and AUFI patients (*p* < 0.001) (Table 1).

The most frequent warning signs were abdominal pain (41/63; 65%), mucosal bleed such as epistaxis, gingivorrhagia, metrorrhagia and hematuria (22/63; 35%), increased hematocrit concurrently with decrease in platelet count (17/63; 27%) and clinical fluid accumulation (6/63; 10%). Two cases were classified as severe dengue: a 29-year-old woman with severe hepatitis and another 36-year-old woman with acute flaccid paralysis.

### 3.2. Type of Infection and Serotype

The dengue patients were investigated for prior dengue infection. Secondary infection was determined in 69.2% of the dengue patients. There was no significant difference in this proportion between the DwoWS (68.2%) and DwWS/SD (71.4%) patients.

The serotype was identified in 85 patients: DENV-4 was the most frequently identified serotype (81/85; 95.3%), but DENV-1 (3/85; 3.5%) and DENV-3 (1/85; 1.2%) were also identified.

### 3.3. NS1 Antigen Levels

Higher values of circulating NS1 antigen were found in DwWS/SD patients (median: 3.5 ng/mL; IQR: 1.7–5.1 ng/mL; *n* = 75) compared to DwoWS patients (median: 2.8 ng/mL; IQR: 1.7–5.0 ng/mL; *n* = 27), although this difference was not statistically significant. However, significantly lower values of circulating NS1 antigen (*p* = 0.01) were found in patients with secondary infections (median: 2.2 ng/mL; IQR: 1.5–4.3 ng/mL; *n* = 66) compared to patients with primary infections (median: 4.6 ng/mL; IQR: 2.5–5.1 ng/mL; *n* = 27) in patients with 4–7 days of disease.

### 3.4. Hematological and Biochemical Parameters

We observed that DwWS/SD patients had significantly lower counts for leukocytes (*p* < 0.001), neutrophils (*p* < 0.001), lymphocytes (*p* = 0.001), monocytes (*p* = 0.001) and platelets (*p* < 0.001), along with elevated levels of ALT (*p* < 0.001) and AST (*p* = 0.002), compared to AUFI and DwoWS patients on admission (Table 1).

In addition, DwWS/SD patients had significantly higher APRI values (*p* < 0.001) compared to DwoWS patients, and significantly higher APRI values were also found in patients with abdominal pain (*p* = 0.001) and mucosal bleeding (*p* = 0.001). Likewise, DwWS/SD patients had significantly lower PLR values (*p* = 0.018) compared to DwoWS patients, but no significant differences were found in relation to NLR and MLR (Table 1).

### 3.5. Differential Immune Mediators Profiles in Dengue Patients

#### 3.5.1. Cytokines and Chemokines

The circulating levels of cytokines and chemokines in Brazilian patients infected with DENV-1, DENV-2 and DENV-3 serotypes were determined previously by our group [19,20,21,22,23]. In the same way as previously, we now characterized the cytokine and chemokine profiles from patients during the dengue-4 epidemic using a multiplex-microbead and ELISA immunoassays.

Pro-inflammatory cytokines were the dominant signature in patients during the dengue-4 epidemic. As in our previous studies, the dengue patients had high plasma levels of the cytokines TNF-α (*p* < 0.001), IL-6 (*p* = 0.005), IL-10 (*p* < 0.001) and, IL-18 (*p* = 0.001) and the chemokines CXCL8/IL-8 (*p* < 0.001), CCL2/MCP-1 (*p* < 0.001), CXCL10/IP-10 (*p* = 0.001) compared to healthy controls (Table 2).

The dengue patients with mucosal bleeding (*n* = 22) presented significantly increased plasma levels of IL-6 (median: 20 pg/mL; IQR: 11.4–38.8; *p* = 0.005) and IL-10 (median: 4.1 pg/mL; IQR: 2.7–11.8; *p* = 0.028) compared to those without mucosal bleeding. Additionally, the dengue patients with thrombocytopenia (<100,000/mm^3^) had significantly higher levels of IL-10 (median = 4.0 pg/mL; IQR: 2.4–9.3; *p* = 0.008) compared to those with platelet counts ≥100,000/mm^3^.

**Figure 1:**
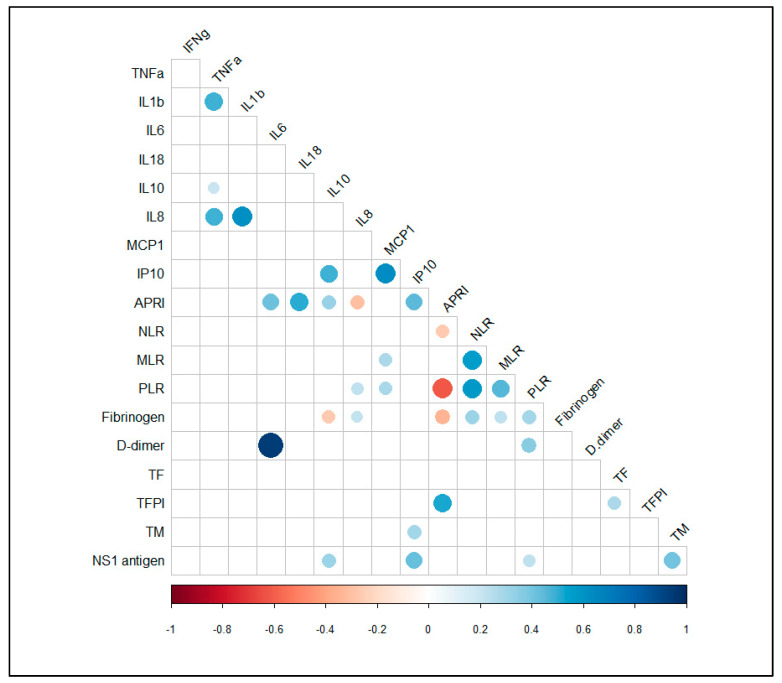
Spearman correlation correlogram between cytokines/chemokines, coagulation parameters and other biomarkers of confirmed cases of dengue. The strength of the correlation between two variables is represented by the color of the circle (only shown if *p* value < 0.05). Colors range from bright blue (strong positive correlation; r_s_ = 1.0) to bright red (strong negative correlation; r_s_ = −1.0).

In addition, we next investigated any association between laboratory parameters and the circulating levels of cytokines/chemokines in the dengue patients. Our analysis revealed that there were positives correlation between plasma levels of IL-18 and transaminases [AST (r_s_ = 0.608; *p* = 0.002; *n* = 23) and ALT (r_s_ = 0.609; *p* = 0.002; *n* = 23)]. Additionally, we found positives correlation between NS1 antigen levels and CXCL10/IP-10 (rs = 0.422; *p* = 0.006; *n* = 41) and between APRI values and IL-6 (r_s_ = 0.415; *p* = 0.003; *n* = 48), IL-18 (r_s_ = 0.502; *p* = 0.015; *n* = 23) and CXCL10/IP-10(r_s_ = 0.442; *p* = 0.002; *n* = 45), respectively (Figure 1).

#### 3.5.2. Coagulation Mediators

Our group has shown coagulopathies during DENV infection in previous epidemics [26,32,33]. In this context, we described some aspects of coagulation and fibrinolysis in patients during the dengue-4 epidemic. In addition to thrombocytopenia, the dengue patients also presented coagulopathy, as demonstrated by increased D-dimer levels (*p* = 0.01) and TFPI plasma levels (*p* = 0.04) and decreased fibrinogen levels (*p* = 0.04) and TF plasma levels (*p* = 0.005) (Table 2). Coagulopathy also contributed to the presence of bleeding: prolonged aPTT was seen in a high proportion of DwWS/SD patients (78%; *p* = 0.004) (Table 1).

The relationship between laboratory parameters and circulating levels of coagulation mediators was analyzed. A positive correlation was found between APRI values and plasma levels of TFPI (r_s_ = 0.530; *p* < 0.001; *n* = 42) and between plasma levels of IL-6 and D-dimer (r_s_ = 0.943; *p* = 0.005; *n* = 6) (Figure 1). Additionally, we found a positive association between plasma levels of TFPI and TF (r_s_ = 0.688; *p* = 0.003; *n* = 16) in DwWS/SD patients.

#### 3.5.3. Principal Component Analysis (PCA) for Cytokines, Chemokines and Coagulation Mediators in Dengue Patients

**Figure 2:**
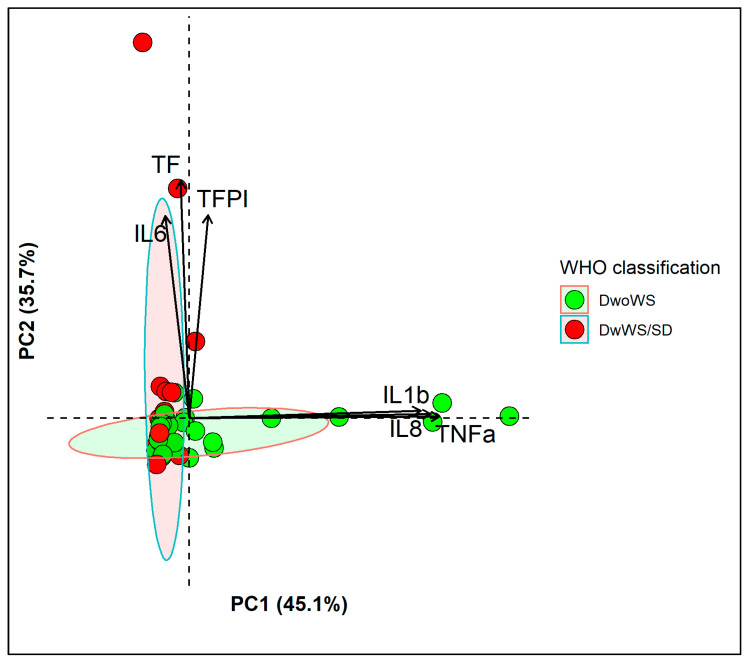
Cytokines/chemokines and coagulation mediator profiles in dengue patients. PCA biplot shows the two principal components that represent a cumulative percentage of variance of 80.8%, PC1 (45.1%): TNF-α, IL-1β and PC2 (35.7%): IL-6, TF and TFPI. Individual scores for each PC are represented as colored points that are grouped according to WHO 2009 classification. (Ellipses represent 95% confidence interval for each group). Loadings of variables are represented as arrows; the length of each arrow represents the contribution of each variable on each principal component.

PCA was performed for cytokines, chemokines and coagulation mediators from 54 dengue patients (39 DwoWS and 15 DwWS/SD). Two different combinations of inflammatory mediators were identified (eigenvalue-one criterion) with a cumulative percentage of variance of 80.8% after varimax rotation. The KMO test result was 0.59, and Bartlett’s test was significant (*p* < 0.001). The pro-inflammatory cytokines TNF-α and IL-1β and the chemokine CXCL8/IL-8 contributed mainly to the first principal component (PC1), while the coagulation mediators TF and TFPI and the pro-inflammatory cytokine IL-6 had an orthogonal contribution to the second principal component (PC2) (Figure 2). For PC1, significantly higher individual score values were observed among DwoWS patients than among DwWS/SD patients (*p* = 0.015). However, for PC2, there was no significant difference between these two groups of dengue patients.

### 3.6. Assessment of Severity Biomarkers

In the univariate analysis, more than 3 days of illness (OR = 3.7; 95% CI 1.9–6.9; *p* < 0.001), hematocrit > 50% (OR = 4.4; 95% CI 1.02–19.07; *p* = 0.048) and high values of APRI (OR: 1.09; 95% CI 1.03–1.15; *p* = 0.002) and IL-6 (OR: 1.09, 95% CI 1.01–1.18; *p* = 0.02) were associated with severity (DwWS/SD patients). In the multivariate analysis, only APRI values were independently associated with severity (adjusted OR: 1.33; 95% CI 1.03–1.71; *p* = 0.027). Multicollinearity was assessed by calculating the variance inflation factor (VIF), and no problem was detected (mean VIF = 1.01).

In the ROC curve analysis, APRI performed better for distinguishing DwWS/SD patients compared to the other factors that were also associated with severity (Table 3).

**Figure 3:**
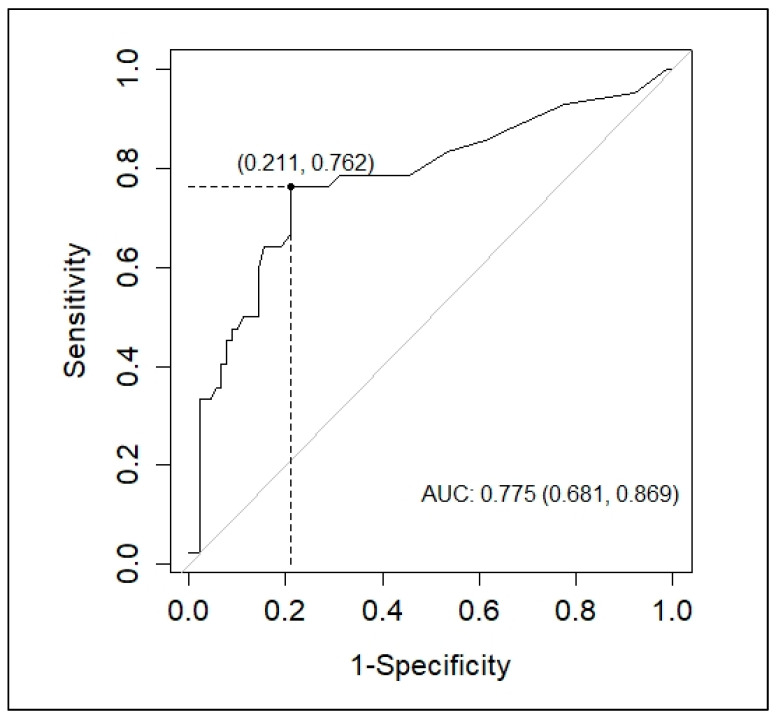
Receiver operating characteristic (ROC) of aspartate aminotransferase-to-platelet ratio index (APRI) for distinguishing DwWS/SD patients. The optimal cutoff point was determined using the Youden index.

An AUC value of 0.775 (95% CI 0.681–0.869) and an optimal cutoff value calculated as 1.4 (sensitivity: 76%; specificity: 79%) were calculated for APRI (Figure 3).

## 4. Discussion

The increased inflammatory response is considered to be the main factor responsible for the pathogenesis of dengue. Therefore, the components of the immune response, including cytokines and other cellular mediators, have been studied as biomarkers of severe disease [34].

This study was conducted in two cities in the regions most affected by the dengue epidemic of 2013 in Brazil (southeastern and central-western regions). DENV-4 and secondary infections were more frequently identified, as had previously been seen in another study conducted during this epidemic [35].

Although it has been established that the circulating levels of NS1 antigen are positively correlated with DENV viremia [36] and are associated with severity [37,38], there is still debate about its usefulness as a biomarker [39]. In our study, no significant difference was found in the circulating levels of the NS1 antigen between the dengue patient groups.

The hematological and biochemical parameters that we determined among dengue patients were consistent with what has been reported in studies of dengue epidemics with the other serotypes [40,41]. The dengue patients, especially DwWS/SD patients, had greater leukopenia, greater thrombocytopenia and higher elevated transaminase levels than the AUFI patients. Although it has been established that liver aminotransferases are not a good biomarker to discriminate severity [42], a recent meta-analysis suggested that the monitoring of platelet counts and transaminase levels during the acute phase of the disease could improve the early prediction of severe dengue [43].

The mechanisms involved in thrombocytopenia during DENV infection are not yet fully understood. Various mechanisms have been hypothesized, including the suppression of bone marrow and the peripheral destruction of platelets, but it has already been established that platelets participate in the inflammatory and immune response [44]. In the present study, the dengue patients with thrombocytopenia had significantly higher levels of IL-10. This finding was in line with the hypothesis of formation of monocyte-platelet aggregates as a cause of thrombocytopenia and cytokine release such as IL-10 [45].

Liver involvement is common in DENV infection and is shown by increased levels of transaminases [46]. Many factors are thought to contribute to liver damage, including hepatocyte apoptosis directly due DENV, hypoxia due to impaired perfusion and immune-mediated injury via upregulated cytokines/chemokines [47]. In our study, it was shown that the transaminase levels of the dengue patients had positive correlations with the plasma levels of IL-18.

The inflammatory response is essential for resistance to DENV infection, and it has been established that this response differs between mild and severe cases [48]. Thus, the cytokine/chemokine profile can serve as a laboratory tool for predicting severe disease [49,50]. In our study, higher plasma levels of the proinflammatory cytokines TNF-α, IL-6 and IL-18, and the anti-inflammatory cytokine IL-10 and the chemokines CXCL8/IL-8, CCL2/MCP-1 and CXCL10/IP-10 were found in the dengue patients compared to the healthy controls. Furthermore, PCA was performed to reduce the dimensionality of the data on the inflammatory and coagulation mediators studied and allowed us to identify two patterns in dengue patients. The first pattern was hyper-inflammatory and was characterized by increases in the levels of the cytokines TNF-α, IL-1β and CXCL8/IL-8, such that they became significantly higher in DwoWS patients. The second pattern was characterized by increases in the levels of the inflammatory cytokine IL-6 and the coagulation mediators TF and TFPI but which did not differ between the two groups of dengue patients, probably due to the fact that the majority were infected with DENV-4 and there were few cases of severe dengue. These findings are consistent with a Thai study that evaluated the cytokine profile in children with subclinical and symptomatic DENV infection and the identification of protective and pathological immunological profiles [24].

Thus, it has been hypothesized that TNF-α, which was found at significantly higher levels in DwoWS patients, belongs to the protective immunological profile, probably due to its ability to exert a protective effect on virus-induced, monocyte-derived dendritic cell apoptosis [51], while the findings on IL-1β and CXCL8/IL-8 could be associated with the predominant DENV-4 serotype and further studies are required. In addition, IL-6, which belongs to the pathological immunological profile and was found at significantly higher levels in DwWS/SD patients, has been associated with severe dengue in several studies [18].

In agreement with our previous studies [19], another pro-inflammatory cytokine that increased markedly in the dengue patients in our study was IL-18. DENV infection activates the NLRP3 inflammasome in macrophages and causes a release of IL-1β and IL-18 by caspase-1 [52]. Studies have shown that high plasma levels of IL-18 coincide with high levels of IL-18 binding protein (IL-18BP), a natural antagonist of IL-18, and that blocking IL-18 activity results in lower IFN-γ levels and higher TNF-α and IL-6 levels [53]. In our study, plasma IFN-γ levels were higher in the dengue patients than in healthy controls, although not significantly. On the other hand, in another study, hepatocytes and lymphocytes from the inflammatory infiltrate in the portal tract showed high expression of IL-18 [54]. In our study, a positive association between IL-18 and transaminase levels was observed, which could reflect the acute inflammatory response that occurs in the liver and contributes to liver damage.

Among the chemokines analyzed, elevated levels of CXCL10/IP-10 in the dengue patients were associated with high levels of NS1 antigen. CXCL10/IP-10 plays an important role in the recruitment of activated NK cells and may compete with DENV for binding to heparan sulfate on hepatocytes. Thus, high levels of CXCL10/IP-10 may limit the spread of infection [55]. In addition, another recent study showed that there was significant upregulation of CXCL10/IP-10 in secondary infection due to the dengue virus compared to the other cytokines analyzed [56].

In DENV infection, both the coagulation and the fibrinolysis processes are activated, thereby causing alterations to their laboratory parameters [44]. In our study, the dengue patients showed significantly elevated levels of fibrinogen, D-dimer and TFPI and decreased levels of TF compared to healthy controls, but no significant difference in the TM levels was seen. Moreover, more than half of the dengue patients presented prolonged aPTT and PT. Thus, the prolongation of aPTT and PT, together with decreased fibrinogen levels and increased D-dimer levels, suggest that increased procoagulant activity is associated with the activation of fibrinolysis as a response mechanism.

The decrease in fibrinogen levels can be explained by higher consumption due to the procoagulant state but also due to extravasation and decreased hepatic fibrinogen synthesis. However, it has been suggested that fibrinolysis in secondary dengue virus infections is not as prominent as in primary infections [57]. In our study, significantly low levels of fibrinogen, although still within the normal range, were found in DwWS/SD patients compared to DwoWS patients.

Studies have suggested monitoring aPTT, PT and D-dimer as early markers of severity and death [57,58,59]. In our study, prolonged aPTT values were found in DwWS/SD patients compared to DwoWS patients, but no significant differences in PT values or D-dimer levels were found between these two groups. Likewise, patients with mucosal bleeding presented elevated levels of IL-6 and IL-10 compared to those without mucosal bleeding, which is explained by the relationship between coagulopathy and the inflammatory response [60,61].

Among the coagulation mediators analyzed, TF and TFPI together with IL-6 formed the second pattern identified in the dengue patients through PCA analysis. Normally, exposure to membrane-bound TF initiates coagulation after vascular injury. Monocytes and endothelial cells do not express TF but do express it under pathological conditions and after exposure to inflammatory cytokines, thus activating the coagulation cascade through the TF pathway. In addition, TF exerts pro-inflammatory activity through activating PARs (protease-activated receptors), which are expressed in endothelial cells, mononuclear leukocytes, platelets, fibroblasts, smooth muscle cells and other cells. This produces upregulation of pro-inflammatory cytokines and adhesion molecules [44]

Overall, regulation of TF and TFPI in dengue infection has been analyzed by few studies to date. In our study, higher levels of TFPI and lower levels of TF were found in dengue patients than in healthy controls, which could be linked to the higher frequency of DENV-4 infected patients. A previous study published by our group had already found that patients with DENV1 and DENV2 infection had higher TF levels than DENV-4 infected patients [32]. TFPI levels increased in response to elevation of TF levels, and thus a positive correlation was shown between these two mediators in DwWS/SD patients in our study. TFPI is an anticoagulant that acts by inhibiting the TF-VIIa complex and factor X, but also has anti-inflammatory properties, which is why it is being studied for the treatment of infectious and inflammatory diseases [62,63].

In addition, it has been established that D-dimer correlates with the activation of the proinflammatory cytokine cascade in critically ill patients [64]. In our study, it was shown that the plasma levels of IL-6 of dengue patients had a positive correlation with the D-dimer levels. IL-6 promotes coagulation without affecting fibrinolysis, and elevated levels of Il-6 increase the surface expression of TF in monocytes and indirectly through C-reactive protein, an acute-phase protein that is released by hepatocytes after stimulation with IL-6, which also increases tissue factor activity in monocytes [65]. IL-6 and D-dimer have recently been shown to be closely related to severe cases of COVID-19 [66].

Lastly, in the multivariate adjusted logistic regression analysis, only APRI was independently associated with severity (adjusted OR: 1.33; 95% CI 1.03–1.71; *p* = 0.027). In addition, our study found a positive association between APRI values and the levels of pro-inflammatory cytokines, chemokines and coagulation mediators (IL-6, IL-18, CXCL10/IP-10 and TFPI) in dengue patients. Hence, this marker could be useful in the triage of patients who attend primary care facilities. Previous works had already proposed APRI as a marker of severity, however, there is still limited information in this regard [67,68]. However, standardized prospective studies with a larger sample size will be necessary to validate our results.

Our study has some limitations. The sample size and non-random selection of participants may not represent the heterogeneity of the general population. It was not possible to analyze the same number of samples for all laboratory markers, and PCA analysis was performed in only 54 patients, which could cause bias in our analysis. Likewise, due to the cross-sectional design of the study, it was not possible to obtain information on dynamic changes to the laboratory markers through paired samples.

## 5. Conclusions

Brazilian patients with dengue during the DENV-4 epidemic presented activation of inflammatory, anti-inflammatory and coagulation mediators, which were associated with alterations to hematological and biochemical parameters and the presence of warning signs. Two cytokine, chemokine and coagulation mediator profiles were identified: one that was protective, with high levels of TNF-α, IL-1β and CXCL8/IL-8, and another that was pathological, with high levels of IL-6, TF and TFPI. Lastly, APRI could be a useful marker in triage of patients attending primary care centers.

## Figures and Tables

**Table 1:** Baseline characteristics and laboratory parameters of dengue and AUFI cases.

Variable	AUFI	DwoWS	DwWS/SD	*p* ^‡^
Total	91	162	63	
Age (years) *	38 (27–51)	36 (26–49)	36 (23–50)	0.869
Gender				
Female	56 (62)	92 (57)	38 (60)	0.737
Male	35 (38)	70 (43)	25 (40)	
Comorbidity				
Hypertension	43 (47)	32 (20)	11 (17)	0.335
Diabetes mellitus	3 (3)	6 (4)	2 (3)	0.975
Others	6 (7)	8 (5)	1 (2)	0.352
Days with illness *	3 (2–5)	3 (2–5)	5 (3–7)	**<0.001**
Hematological parameters				
Hematocrit ^†^ (%)	42.3 ± 3.7	41.9 ± 4.0	43.2 ± 4.6	0.203
WBC * (×mm^3^)	6090 (3940–7400)	3900 (2860–5300)	3450 (2500–4950)	**<0.001**
Neutrophils * (×mm^3^)	3098 (2184–4641)	1950 (1305–3142)	1476 (972–2250)	**<0.001**
Lymphocytes * (×mm^3^)	1560 (1168–2112)	1272 (861–1716)	1123 (792–1650)	**0.001**
Monocytes * (×mm^3^)	460 (341–680)	386 (301–546)	304 (195–498)	**0.001**
Platelets * (×10^3^/mm^3^)	190 (140–233)	159 (127–211)	93 (43–179)	**<0.001**
Biochemical parameters				
AST * (IU/L)	30 (23–50)	41 (29–69)	93 (40–260)	**<0.001**
ALT * (IU/L)	41 (30–67)	50 (37–80)	75 (43–198)	**0.002**
Coagulation parameters				
Prolonged aPTT (>40 s)	ND	33/68 (49)	28/36 (78)	**0.004**
Prolonged PT (>14 s)	ND	34/69 (49)	21/36 (58)	0.378
Other biomarkers				
APRI *	0.5 (0.3–1.1)	0.7 (0.4–1.2)	2.5 (1.4–19.7)	**<0.001**
NLR *	1.9 (1.4–2.9)	1.7 (1.0–2.5)	1.4 (0.9–2.8)	0.120
MLR *	0.3 (0.2–0.4)	0.3 (0.2–0.4)	0.3 (0.2–0.5)	0.678
PLR *	113.9 (77.8–183.6)	120.7 (84.8–170.7)	91.9 (41.3–164.3)	**0.018**
Dengue diagnosis				
AgNS1 positive	0 (0)	83 (51)	31 (49)	
IgM anti-dengue positive	0 (0)	73 (45)	51 (81)	
RT-PCR DENV positive	0 (0)	71 (44)	14 (22)	

(*) median (interquartile range); (^†^) mean ± standard deviation; (^‡^) Bold values denote statistical significance (*p* < 0.05); DwoWS: Dengue without warning signs; DwWS/SD: Dengue with warning signs/Severe dengue; AUFI: acute undifferentiated febrile illness; WBC: white blood count; AST: aspartate aminotransferase; ALT: alanine aminotransferase; ND: not determined; aPTT: activated partial thromboplastin time; PT: prothrombin time; APRI: aspartate aminotransferase-to-platelet ratio index; NLR: neutrophil-to-lymphocyte ratio; MLR: monocyte-to-lymphocyte ratio; PLR: platelet-to-lymphocyte ratio.

**Table 2:** Cytokines, chemokines and coagulation mediators in healthy controls and confirmed cases of dengue according to WHO 2009 classification.

	Healthy Controls	DwoWS	DwWS/SD	*p* ^‡^
**Cytokines/Chemokines**				
IFN-γ * (pg/mL)	115.3 (80.5–133.7)	306.0 (87.6–400.4)	197.8 (120.8–290.3)	0.383
TNF-α * (pg/mL)	3.1 (2.6–4.4)	7.9 (5.9–11.6)	7.0 (5.0–9.6)	**<0.001**
IL-1β * (pg/mL)	0.5 (0.3–1.2)	0.6 (0.4–1.1)	0.5 (0.4–0.7)	0.109
IL-6 * (pg/mL)	5.1 (3.3–7.7)	8.6 (6.0–13.2)	12.4 (8.7–20)	**0.005**
IL-10 * (pg/mL)	0.4 (0.4–0.6)	2.0 (1.0–4.3)	3.5 (2.1–8.2)	**<0.001**
IL-18 * (pg/mL)	8.9 (8.1–10.3)	17.4 (11.1–26.7)	24.7 (16.1–31)	**0.001**
CXCL8/IL-8 * (pg/mL)	2.3 (1.6–3.5)	9.3 (5.1–22.5)	6.6 (3.5–11)	**<0.001**
CCL2/MCP-1 * (pg/mL)	136.7 (42.9–226.1)	668.9 (336.9–977.9)	545.5 (362.8–979.6)	**<0.001**
CXCL10/IP-10 * (pg/mL)	64.3 (40.8–104.1)	558.7 (180.8–796.2)	618.9 (309.2–779.3)	**0.001**
**Coagulation mediators**				
Fibrinogen * (mg/dL)	296 (224–357)	313 (266–367)	265 (193–334)	**0.037**
D-dimer * (ng/mL)	317 (235–490)	616 (507–852)	620 (421–944)	**0.010**
TF * (pg/mL)	19.4 (15.1–26.7)	10.1 (8.5–13.5)	9.9 (9.4–11.3)	**0.005**
TFPI * (pg/mL)	14117 (10599–22478)	20400 (17250–28225)	20468 (17189–33215)	**0.042**
TFPI/TF *	719.5 (618.1–1720.3)	1914.9 (1100.6–2487)	1848 (1387.7–2671.7)	0.199
TM ^†^ (pg/mL)	3047 ± 763	3227 ± 702	3154 ± 888	0.786

(*) median (interquartile range); (^†^) mean ± standard deviation; (^‡^) Bold values denote statistical significance (*p* < 0.05); DwoWS: Dengue without warning signs; DwWS/SD: Dengue with alarm signs/Severe dengue; TF: Tissue factor; TFPI: tissue factor pathway inhibitor; TM: thrombomodulin; Fibrinogen (normal range: 200–400 mg/dL); D-dimer (normal range: ≤500 ng/mL).

**Table 3:** AUC values obtained from ROC analysis for biomarkers associated with severity.

	AUC	IC 95%		Cut-Off	Sensitivity	Specificity
HCT > 50%	0.533	0.494	0.571	*NA*	8.6%	98%
APRI	0.775	0.681	0.869	1.4	76%	79%
IL-6	0.693	0.562	0.825	7.8	95%	40%

HCT: Hematocrit; APRI: aspartate aminotransferase-to-platelet ratio index; *NA*: not applicable.
